# From Bench to Biomolecular Simulation: Phospholipid Modulation of Potassium Channels

**DOI:** 10.1016/j.jmb.2021.167105

**Published:** 2021-08-20

**Authors:** Tanadet Pipatpolkai, Daniel Quetschlich, Phillip J. Stansfeld

**Affiliations:** 1 Department of Biochemistry, South Parks Road, Oxford OX1 3QU, UK; 2 Department of Physiology Anatomy and Genetics, Parks Road, Oxford OX1 3PT, UK; 3 OXION Initiative in Ion Channels and Disease, University of Oxford, Oxford OX1 3PT, UK; 4 Department of Chemistry, South Parks Road, Oxford OX1 3QZ, UK; 5 School of Life Sciences & Department of Chemistry, University of Warwick, Coventry CV4 7AL, UK

**Keywords:** K^+^ channels, phospholipids, phosphatidylinositol-bisphosphate, protein-lipid interactions, molecular dynamics simulations, electrophysiology, flux assays, mass spectrometry, Super-resolution microscopy, X-ray crystallography, Cryo-EM microscopy

## Abstract

Potassium (K^+^) ion channels are crucial in numerous cellular processes as they hyperpolarise a cell through K^+^ conductance, returning a cell to its resting potential. K^+^ channel mutations result in multiple clinical complications such as arrhythmia, neonatal diabetes and migraines. Since 1995, the regulation of K^+^ channels by phospholipids has been heavily studied using a range of interdisciplinary methods such as cellular electrophysiology, structural biology and computational modelling. As a result, K^+^ channels are model proteins for the analysis of protein-lipid interactions. In this review, we will focus on the roles of lipids in the regulation of K^+^ channels, and how atomic-level structures, along with experimental techniques and molecular simulations, have helped guide our understanding of the importance of phospholipid interactions.

## Highlights

• Interactions of lipids with K^+^ channels.
• Structural basis of PIP_2_ binding.
• Experimental methods for quantifying PIP_2_-binding to K^+^ channels.
• Molecular simulation methods for studying PIP_2_ binding.

## Graphical abstract

**Figure:**
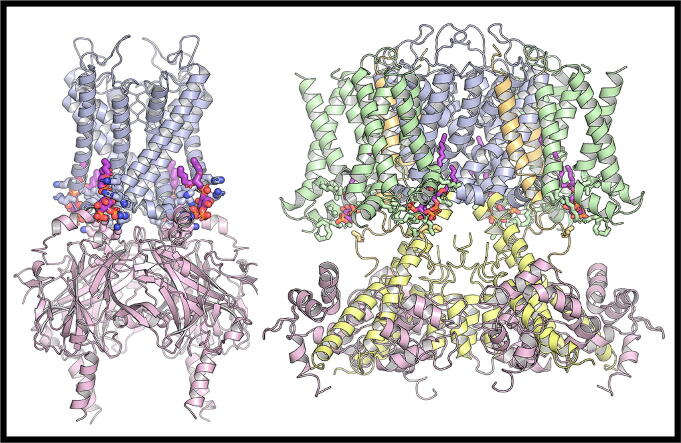

## Introduction

In the mid 1990s, Rebecca Ball and Don Hilgemann identified that a lipid involved in cell signalling, phosphatidylinositol-4,5-bisphosphate (PIP_2_), was also able to directly interact with ion channels.^1^ Since then, multiple methodologies and concepts have been developed to understand the roles for lipids as regulators of ion channels.^2^ The determination of potassium (K^+^) channel structures, and the development of computational tools to study them, have also greatly contributed to our perspectives on the molecular basis of ion channel interactions with lipids.^3^, ^4^, ^5^

K^+^ channels are integral membrane proteins which facilitate the passive movement of K^+^ ions across the membrane. The selective permeation of ions induce ionic gradients that are crucial in cellular communications, metabolism and homeostasis.^6^ Mutations of K^+^ channels cause many clinical symptoms such as cardiac arrhythmia, neonatal diabetes and migraines.^7^, ^8^, ^9^ As K^+^ channels are transmembrane (TM) proteins, one of their key modulators is their lipid environment, with membrane lipids binding both specifically and/or indiscriminately to the proteins.^10^ Mechanistic understanding of protein-lipid interactions is essential to inform research on novel therapeutic agents that may modulate ion channel activity in diseases.^11^

In this review, we will discuss our current understanding of the diversity of K^+^ channels and how these channels are modulated by phospholipids. We discuss how the interactions with lipids impact on channel gating and/or stabilise distinct conformations of the proteins, and review how complementary techniques may be applied, detailing how experimental and computational approaches may be applied to calculate values for protein-lipid binding. Finally, we discuss how knowledge of ion channel gating has shed light on therapeutic approaches to clinical disease.

## **K**^+^**channel diversity**

**Figure 1:**
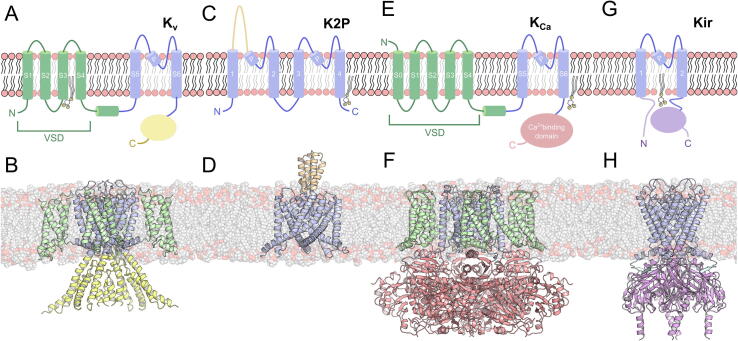
**Topological representation and atomic structures of K^+^ channels. (A)** Voltage-gated potassium (K_v_) channel topology. **(B)** A structural example for of a K_v_ channel: hKCNQ1 (PDB ID: 6V01).^56^**(C)** Two-pore potassium (K_2P_) channel topology. **(D)** Example structure of the K_2P_ channel, mTREK-1 (PDB ID: 6V37). **(E)** Ca^2+^ gated potassium (K_Ca_) channel topology. **(F)** Structure of the K_Ca_ channel hSlo1 (PDB ID: 6V38).^143^**(G)** Inwardly rectified potassium (K_ir_) channel topology. **(H)** Structure of the cK_ir_2.2 channel (PDB ID: 3SPI).^144^ Topology of potassium channels where transmembrane helices are represented as cylinders. The structures are shown in cartoon. The voltage sensor domain (VSD) is coloured green. The pore domains are all represented in blue. The cytoplasmic domain of K_v_, K_Ca_ and K_ir_ are shown in yellow, red and lilac respectively. The cap domain of K_2P_ channel is shown in orange. N and C represent each termini of the protein. The PIP_2_ cartoon represents a putative binding site based on experimental and/or structural data.

K^+^ ions are transported through a central pore domain that is common to all members of the K^+^ channel family (Figure 1). This domain comprises two TM helices linked by a pore (P) half-helix and a highly-conserved ion selectivity filter.^12^ Four copies of the pore topology must be present to form a functional pore, and therefore most K^+^ channels are tetramers. An exception is the 2-pore (K_2P_) family where each monomer has two repeated pore topologies, and therefore requires only a dimer of proteins (Figure 1(C)). K^+^ channels are further functionalised though additional domains that regulate gating of the channel.^13^

Voltage-gated K^+^ (K_v_) channels gate in response to changes in the potential across the membrane. In addition to the canonical pore domain, the topology of a K_v_ channel contains four further TM helices, known as the voltage-sensor domain (VSD)^14^ (Figure 1(A),(B)). The fourth TM helix (S4) contains multiple basic residues that respond to a change in membrane potential, moving towards the extracellular side upon membrane depolarization.^15^, ^16^ This motion of the S4 helix is coupled to the channel pore through an amphipathic helix^17^; as the S4 helix translates, the linker is pulled away from the inner helices of the pore, therefore permitting channel opening.^18^ Conversely, hyperpolarization-activated cyclic nucleotide-gated (HCN) channels, which share a similar TM domain topology, open at hyperpolarized voltages, with the inward motion of the S4 helix resulting in channel opening.^19^, ^20^

K_2P_ channels are denoted by the fact that each monomer has two pore domain sequences (Figure 1(C),(D)). The regulation of K_2P_ channels differs between their members, and can involve responses to changes in pH (TASK^21^) and/or membrane pressure (TREK1, TREK2 and TRAAK).^22^, ^23^ The diversity and mechanisms of these proteins is discussed in more detail as part of this series of ion channel reviews.^4^

Calcium regulated K^+^ (K_Ca_) channels are expressed ubiquitously throughout the human body and their single-channel conductance is the highest of all K^+^ channels.^24^ K_Ca_ channel opening is induced by an increase in intracellular concentration of Ca^2+^; with the divalent ion binding to the C-terminal domain of the K^+^ channel, as the intracellular ionic concentration rises from the nM to μM range. These channels also have a VSD, akin to K_v_ channel, and are therefore also voltage sensitive (Figure 1(E),(F)).^25^ This makes the channels activatable by both Ca^2+^-binding and membrane depolarization. In addition to the tetrameric pore, K_Ca_ can have auxiliary subunits which are tissue specific and can modify the Ca^2+^ sensitivity of the channel as well as gating properties.^26^

Inwardly rectifying K^+^ (K_ir_) channels conduct current at a negative potential, and cease to do so as the membrane become more depolarised, being inhibited by direct channel block by polyamines and divalent cations.^27^, ^28^ There are several publications that review the structures, regulation and function of K_ir_ channels.^29^, ^30^ K_ir_ channels generally have a short, 50–90 amino acid, N-terminal domain, and a larger C-terminal domain comprised of up to ~ 250 residues (Figure 1(G),(H)). These channels associate with other proteins to form larger complexes and are regulated by several factors.^31^ For example, K_ir_3.x channels are activated by the binding of G_β_ and G_γ_ subunits from the G_i/o_ proteins.^32^ On the other hand, K_ir_6.2 associates with 4 regulatory sulfonylurea receptor (SUR) subunits^33^, ^34^, ^35^ to form the ATP-sensitive K (K_ATP_) channel, and is inhibited by the binding of ATP to the pore subunits of the channel.^36^

## Identifying a functional role for PIP_2_ binding to K_ir_ channels

Many K^+^ channels are functionally modulated by specific interactions with lipids from the surrounding membrane. Lipids, in particular PIP_2_, were first identified as channel activators of the ATP-sensitive K^+^ (K_ATP_) channel.^1^, ^37^, ^38^ Here, an increase in levels of PIP_2_ – by either activation of phospholipase C (PLC) or direct application of PIP_2_ – was shown to reduce channel inhibition by ATP.^1^, ^37^, ^38^ This could either be due direct binding site competition between ATP and PIP_2_ or allosteric modulation of the ligand-induced opening and closing events.^39^, ^40^

Application of PIP_2_ to the K_ir_1, K_ir_2, K_ir_3 and K_ir_6 channels has been shown to increase the size of the current of an excised patch and also elevates their open probability (P_open_).^41^, ^42^, ^43^ To evaluate the response of the channel to PIP_2_ and to characterise the residues involved in coordinating PIP_2_, the size of the current after patch excision has been used as a readout.^45^ Mutations which decrease PIP_2_ binding to the K_ir_6.2 channel also reduce channel burst time duration, and result in a faster *run-down*; where the current flowing through the channel in an excised patch declines over time.^46^ The molecular mechanism of the *run-down* has been proposed to be associated with PIP_2_ disassociating from its binding site in the excised patch, or a depletion in the local concentration of PIP_2_, which ultimately causes the channel to shut.^47^ One method to reduce the level of *run-down* is to apply a fluoride vanadate and pyrophosphate (FVPP) solution, which inhibits the degradation of PIP_2_ by phosphatases.^48^ Together, this increases the local PIP_2_ concentration in the patch and enhances PIP_2_-channel interactions in the excised patch.^49^ A second method used to qualitatively assess channel activation by PIP_2_ is to introduce non-native voltage sensitive phosphatases (VSPs) to the cell of interest.^50^ This has been shown to be more effective than activation of PLC by Ca^2+^ due to a greater selectivity and specificity to the PIP_2_ in the membrane. These studies suggest that PIP_2_ is involved in stabilising the channel in its open conformation, as well as initially promoting channel opening. Interestingly, PIP_2_ binding to the K_ir_ channel homologue (K_ir_Bac1.1) closes the channel, despite the absence of PIP_2_ in bacterial membranes.^44^

## Regulation of other K^+^ channels by PIP_2_

Voltage gated K^+^ (K_v_) channels, including KCNQ1 (K_v_7.1),^5^, ^51^ hERG (K_v_11.1),^52^ Shaker (K_v_1.2)^53^ and K_v_2.2,^54^ are primarily gated by voltage but can also be modulated by PIP_2_. Similar to K_ir_ channels, application of PIP_2_ to the KCNQ1 and hERG channels slows channel *run-down*.^58^, ^59^ For KCNQ1, three binding sites have been proposed; one on the VSD, one on the linker between the VSD and pore domain, and one directly adjacent to the pore.^55^, ^56^, ^57^ The multiple binding sites therefore means that modulation of KCNQ1 by PIP_2_ is complex.

PIP_2_ is absolutely necessary to bridge the intramolecular interactions between the S4 helix of the VSD domain,^60^ this therefore enhances the VSD coupling to the pore.^53^, ^61^ PIP_2_ also appears to induce a large scale conformational change of the intracellular domain of the channel, which is also important for channel gating.^56^ In addition, PIP_2_ also has a dynamic interplay with other KCNQ1 regulator such as calmodulin (CaM).^62^

Application of PIP_2_ directly to K_2P_ channel family members, including TREK-1, TASK1, TASK3 and TRAAK channels, increases the size of the current influx and modulates voltage dependency after tension is applied to the bilayer.^63^, ^64^ The binding of the PIP_2_ headgroup to TREK1 exhibits bimodal activity, with channel activation observed at low concentrations and inhibition at higher concentrations.^65^

K_Ca_ channels are also sensitive to PIP_2_, with structural studies revealing a KDRDD-loop to be essential for the interaction.^66^ While PIP_2_ may activate K_Ca_ channels alone, the primary effect of PIP_2_ binding has been shown to be enhancement of Ca^2+^ induced gating of the channel.^67^ PIP_2_-K_Ca_ channel interactions also depend on lipid tail length, subunit stoichiometry and tissue type.^67^, ^68^

## Roles for other anionic phospholipids and cholesterol in modulating ion channel activity

Anionic phospholipid such as phosphatidylglycerol (PG) have been shown to provide a secondary mode of regulation for ion channels such as K_ir_2.1 channel.^69^, ^70^, ^71^ This regulation includes tuning the channel activation by PIP_2_, e.g. in K_ir_2.2 channels where PIP_2_ activation is enhanced by approximately 100-fold when PG, PS, PA, and PI are also present.^69^, ^71^ Based on the negative electrostatic charge of the headgroup, it could be proposed that the anionic lipids, e.g. PS and PG, compete with PIP_2_ binding for the primary binding site.^72^ For the K_ir_2.2 channel, PS has been shown to have a second binding site, adjacent to the primary PIP_2_ binding site. The affinity of PS for K_ir_2.2 increases when PIP_2_ is bound.^73^ Other inositol lipids, including PI4P and PI, have also been shown to activate K_ir_ channels, but to a lesser extent.^74^, ^75^ Conversely, for PIP_3_, the additional phosphate group appears to reduce binding to the TREK1 channel.^72^ Therefore, channel activation by PIP lipids is not driven solely by charge.

Modulation of K^+^ channel activity by anionic phospholipids has been demonstrated directly through the application of the lipids to a patch,^76^ or by enzymatic activation such as phospholipase D2 (PLD2) and therefore generating a local intracellular anionic phospholipids.^77^ An example of a direct activator includes intracellular lysophosphatidic acid, which has been previously shown to directly activate TREK1, TREK2 and TRAAK channels.^76^ Phosphatidic acid (PA) has also been shown to activate a chimeric K_v_ channel (voltage sensor domain of K_v_2.1 fused with a K_v_1.2 pore domain).^78^ On the other hand, indirect activation mediated through PLD2 produces multiple products such as phosphatidic acid (PA), phosphatidylglycerol (PG) and phosphatidylethanol (PEtOH). These lipids have been previously suggested to activate multiple K^+^ channels such as K_ir_, TREK1 and TRAAK.^79^, ^80^, ^81^

In addition to PIP and other anionic lipids, cholesterol has also been shown to modulate a subset of K^+^ channels. Several reviews have been dedicated to the regulation of K^+^ channels by cholesterol.^82^, ^83^, ^84^ Cholesterol has been shown to inhibit K_ir_1.1, K_ir_2.2 and K_ir_6.2 channels by binding to the CD loop between the transmembrane domain and the cytoplasmic domain^84^ and closing the channel.^85^ Curiously, closure of K_ir_ channels by cholesterol is independent of channel activation by PIP_2_.^85^ Overall, this suggests that cholesterol stabilises the closed state of the channel as it binds to the CD loop, rather than triggering a competitive gating mechanism, or competing for the PIP_2_ binding site.

## Crystal structures highlight lipid binding sites and conformational change

**Figure 2:**
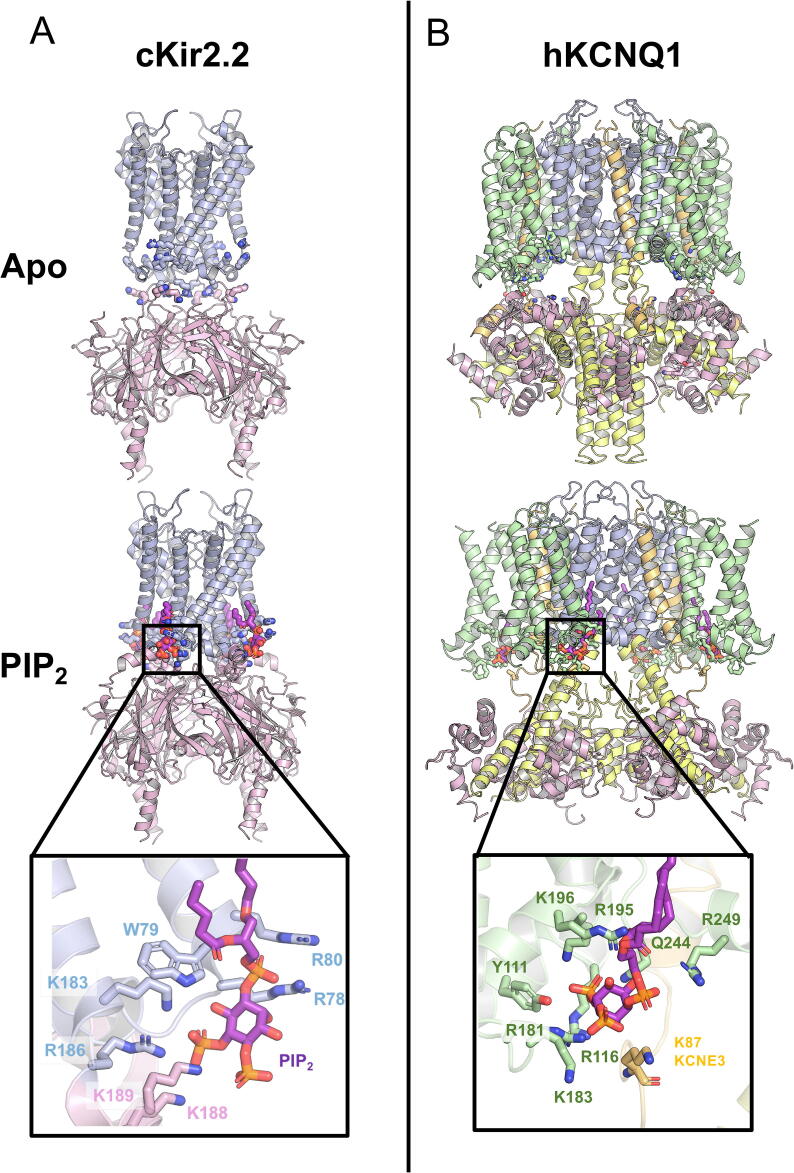
**PIP_2_ binding site on cK_ir_2.2 and hKCNQ1 channel. (A)** Atomic structure of the cK_ir_2.2 channel in Apo (PDB ID: 3JYC)^145^ and PIP_2_-bound state (PDB ID: 3SPI).^87^ The pore domain is shown in blue and the cytoplasmic domain is shown in pink. **(B)** Atomic structure model of KCNQ1 channel in KCNE3-CaM bound (PDB ID: 6V00) and KCNE3-CaM-PIP_2_-bound state (PDB ID: 6V01).^56^ The pore domain is shown in blue, voltage sensor domain is coloured in green, S6 helices in the cytoplasm are shown in yellow, calmodulin proteins are shown in pink and KCNE3 subunits are shown in orange. In both panels, carbon atoms on PIP_2_ are represented in purple and others are coloured based on CPK convention. All PIP_2_ bound residues are shown as sticks.

In 2011, X-ray structures of K_ir_2.2 and GIRK (K_ir_3.2) channels revealed well-defined densities for PIP_2_.^86^, ^87^ These structures highlight the key amino acid residues coordinating the bound PIP_2_ lipid, as well as showing how PIP_2_ induces conformational changes and drives opening of the channel gate. The PIP_2_ binding site on a K_ir_ channel is predominantly coordinated by basic residues (lysine and arginine), which engage with the phosphate groups (Figure 2(A)). These residues are located on both N-terminal and C-terminal domains of the protein, with the binding site being formed between adjacent subunits. At the N-terminal end of TM1 of K_ir_2.2, residue W79 engages with the inositol sugar ring and lipid tails, while R78 and R80 form hydrogen bonds with the 1′ phosphate. In the C-terminal domain, K183, R186 and K188, of the C-linker, interact with the 5′ phosphate and K189 coordinates both 4′ and 5′ phosphates.^87^

Conformational changes of the K_ir_ channels were proposed by comparing the bound and apo states of the structures. The binding of PIP_2_ to a K_ir_ channel introduces a 6 Å upward conformational change and rotation of the C-terminal domain (Figure 2(B)). Similar structural changes have also been observed in the bacterial K_ir_ channel, K_ir_Bac.^88^ This twists the TM helices of the pore and widens the distance at the I177 constriction point from 4.9 Å to 6.3 Å. However, this alone is insufficient to hydrate the lower gate of the pore, in part due to its hydrophobicity.

Capturing ion channels in different states has been a major technical challenge. Despite the wealth of information gleaned from X-ray crystallography, structures are generally captured at stable energy minima and thus transient intermediates are difficult to trap. This has been a major a bottleneck for studying the subtleties of lipid-dependent conformational change.

## The Cryo-EM revolution and protein-lipid interactions

A major breakthrough for protein-lipid interaction studies has been the improvement to cryo-electron microscopy (cryo-EM) over recent years. This has yielded numerous atomic and near-atomic resolution structures of membrane proteins solubilised in detergents and nanodiscs. As a result, both novel structures and states have been observed, with many cryo-EM structures of membrane proteins revealing lipids, including phosphatidylglycerol (PG), cholesterol, PIP_2_ and phosphatidylinositol (PI), bound to the structures.^10^

Recent cryo-EM structures of the KCNQ1 and K_ir_3.2 channels have captured binding sites for PIP_2_. The quality of the electron microscopy density map allows precise identification of interactions between the lipid phosphate groups and the basic side chains. For the structures of the KCNQ1 channel, the PIP_2_ binding site is shown to be located on the VSD, adjacent to the S2-S3 linker (Figure 2(B)).^56^ In comparison to the apo-state structure, the PIP_2_ binding appears to induce a large conformational change in the S6 helix, as the helix bends at the RQKH motif. This causes the S6 helix and C-terminus of the protein to splay outwards and widens the cavity of the pore (Figure 2(B)).

Multiple cryo-EM structures of the K_ir_3.2 channel illustrate how PIP_2_ mediates an interaction between K_ir_3.2 and the α-subunit of a G-protein.^89^ Using cryo-EM, the study titrated in increasing concentrations of PIP_2_ to trap different states of K_ir_3.2. Similar to the chicken K_ir_2.2 structure, an upward motion of the C-terminal domain by approximately 6 Å is observed when PIP_2_ is bound to the channel. This conformational change shifts the position of the βL-βM loop upwards, allowing G_βγ_ to bind to the channel and triggers an opening event. In contrast to other models of regulation, the binding of G_βγ_ to the channel may only happen after the PIP_2_ binding event.^89^

## Membrane embedding ion channels and identifying lipid binding sites with MD simulations.

Molecular dynamics (MD) simulations enable the unbiased prediction of lipid binding sites to ion channels within a membrane environment.^90^ Due to limits in simulation time and slow diffusion of lipids, coarse-grained (CG) MD simulations are regularly applied to study lipid binding. These simulations usually commence with lipids unbiasedly placed around an ion channel, either as a preformed bilayer or in random orientations to allow membrane self-assembly. As part of the simulation the lipids and protein are allowed to freely diffuse, with specific lipid-protein interactions recorded as the simulation proceeds.^73^, ^91^ From these simulations the nature of the binding site can be quantified by several factors, such as residue-lipid contacts, the relative residence time for the lipid within the binding site and membrane deformation around the protein.^73^ These methods have been shown to be have both excellent agreement with structurally determined sites^73^ and also for proposing uncharacterised binding sites.^91^ Therefore, these methods have the potential to identify bounds lipids based on unassigned electron density.^92^ In the future, this could be integrated into structural processing software to aid identification of a lipid at its site.

In addition to the PIP_2_ binding sites observed in the cryo-EM structure of the KCNQ1 channel, electrophysiology studies have proposed that PIP_2_ binds to multiple intracellular loops, the VSD and the pore.^55^, ^93^, ^94^, ^95^, ^96^ MD simulations with PIP_2_ bound at these sites have shown that the lipid bridges the VSD to the pore, and thereby enhances their coupling.^60^ MD simulations may therefore also be used to highlight potential gating mechanisms of an ion channel, induced by bound lipids. Understanding the residues involved in the conformational changes aids characterisation of the clinical phenotypes at the molecular level. This could potentially be used to probe clinical symptoms and outline potential therapies.

## Computational tools to investigate the strength of protein-lipid interactions

With improvements to both computational hardware and the software for molecular simulation, longer timescale and/or larger simulations can be performed to identify lipid binding sites.^73^ Once a lipid-binding site is identified, the natural next step is to calculate binding free energy. However, all-atom computational binding free calculations can be highly costly due to convergence and sampling issues of lipids within a membrane environment. Therefore, CG approaches have been applied to overcome the limitations of, e.g., diffusion at an atomistic-level.^97^, ^98^ To date, a number of computational approaches have been used to identify lipid binding affinity, for a recent review on this topic see.^98^

**Figure 3:**
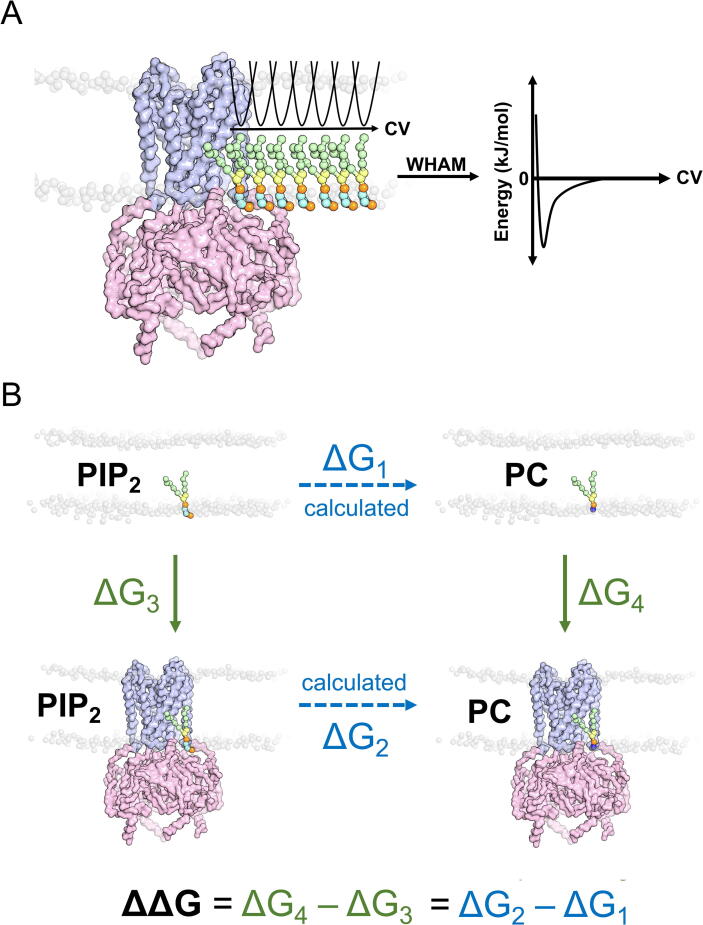
**Computational methods associated with K^+^ Channel lipid interactions. (A)** Potential of mean force (PMF) calculation. PIP_2_ lipids (acyl tail - green, glycerol - yellow, phosphates - orange, inositol ring - cyan) are sampled with a harmonic potential along the collective variable (CV) parallel to the bilayer. Each PIP_2_ represents a sampling position. The protein is displayed only in coarse-grained backbone where the transmembrane region is shown in blue and cytoplasmic region is shown in pink. PIP_2_ binding free energy to the protein is then calculated using weighted histogram analysis method (WHAM) based on the energies it samples along the CV (right). **(B)** The thermodynamic scheme underpinning free energy perturbation (FEP) calculation. Transformation of PIP_2_ to PC in both bound and free configurations are calculated in ΔG_1_ and ΔG_2_ (blue). This allows ΔG_3_ and ΔG_4_ to be obtained (green) using the equation shown (bottom).

One method is to use Potential of Mean Force (PMF) calculations to measure the free energy of lipid interactions from bound to unbound, along what is referred to as a collective variable (CV) (Figure 3(A)). By using umbrella sampling, lipids may be simulated at fixed, incremental distances from the protein binding site.^99^ This yields a calculated lipid binding free energy profile from bound to unbound, whilst also providing a total free energy difference between the bound and unbound states. The first attempt of PMF calculations to calculate protein-lipid affinity was for cardiolipin binding sites on the cytochrome c oxidase and complex IV.^100^, ^101^ Since this initial approach, the method has been applied to study lipid binding affinity to receptors, transporters and ion channels.^92^, ^97^, ^99^, ^102^, ^103^ The method has also shown to be useful when comparing differences in the affinities for a range of lipid types and provides details of their 1D energy landscape. There are other CV-based enhanced sampling methods that can also be used to calculate binding free energies across multiple dimensions, e.g. well-tempered metadynamics.^99^

A second approach for quantifying lipid binding affinity is free energy perturbation (FEP). FEP is often referred to as “alchemical transformation”, where molecules or subgroups of molecules are computationally mutated from one to another. From this transformation a relative binding free energy between the bound and free states can calculated^104^ (Figure 3(B)). Recent development of CG-FEP approaches illustrates strong agreement with the calculations using PMF approaches, but with a reduced computational cost.^99^, ^105^ The method can also be applied to amino acids to investigate the effects of site-directed mutagenesis on the binding free energy^105^, with the potential to develop this into a high-throughput screen for e.g. ion channel mutations that cause disease, which would be tightly coupled to experimentally determined measurements.

## Experimental affinity, gating and efficacy of lipids

There are several approaches that have been used to quantify lipid binding affinities and the extent of channels activation by lipids both *in vitro* and *in vivo*.^2^, ^106^ In this section, we will discuss several experimental methods which could be used to measure the extent of channel activation/binding by PIP_2_.

**Figure 4:**
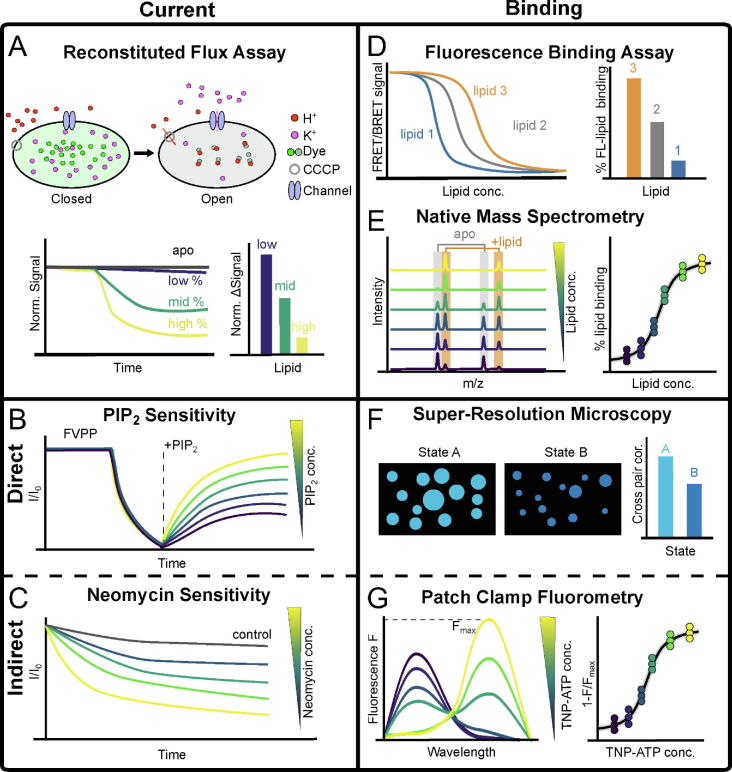
**Experimental methods associated with K^+^ channel lipid interactions. (A)** Reconstituted flux Assay. Schematic diagram showing the fluorescence properties of the liposome based on.^72^ The liposome contains K^+^ and fluorescence sensitive dye (green). Upon an addition of CCCP - uncoupler (grey circle) and the opening of the channel (blue), K^+^ ions (pink) move out of the liposome, counteracting with the movement of proton (red) into the liposome. The protons then quench the fluorescence signal (green to grey). The degree of quenching is depending upon the concentration of the channel activator. **(B)** PIP_2_ sensitivity. The FVPP labels its application to prevent rundown. The channel is then allowed to rundown almost to 0 through the wash-out of FVPP. PIP_2_ at different concentrations (low - dark blue, high - yellow) are applied to the patch to observe an increase in channel current. The y-axis is labelled as the fraction of the maximal current. Neomycin sensitivity. **(C)** Neomycin at different concentrations (low - dark blue, high - yellow) are applied to the patch to observe decrease in channel current as PIP_2_ is quenched from the membrane. **(D)** Fluorescence binding assay. Decrease in fluorescence emission in an *in vitro* binding assay caused by FRET as different lipids (shown in different colour) compete with a PIP_2_ analogue at the binding site. Relative change in fluorescence recorded by the assay. **(E)** Native mass spectrometry. The traces are in different colours to show the influence of PIP_2_ concentration (low - dark blue, high - yellow), when applied to the protein in a native membrane or as a purified protein. Relative peak height can be used to calculated PIP_2_-bound saturation curve. **(F)** Super-resolution microscopy. Two states of the cells (A and B) display different sizes of lipid clustering observed under fluorescence microscope. Size of the cluster can be calculated to infer degrees of protein-lipid interaction. **(G)** Patch-clamp fluorometry. Fluorescence emission detected at specific wavelength. Different TNP-ATP concentrations (low - dark blue, high - yellow) are applied to a patch, resulting in a shift in the fluorescence emission peak caused by FRET. Relative fluorescence measured from the panel on the left are used to calculate TNP-ATP saturation curve.

Ion flux across the ion channels may be measured in reconstituted systems or in intact cells. Full details for configuring reconstituted system have been reviewed previously.^107^, ^108^, ^109^ Ion flux assays may be performed using K^+^ channel permeant ions such as Rb^+^ or Tl^+^ and/or fluorescence dyes.^109^ For example, K^+^ channels may be reconstituted into liposomes containing a Tl^+^-sensitive fluorescence dye and K^+^ ions. When the liposome is placed in an extracellular Tl^+^ solution, the Tl^+^ enters the liposomes through the K^+^ channels, and K^+^ diffuses out; with both ions permeating down their concentration gradients. The increased internal concentration of Tl^+^ quenches the fluorescence and thus provides a signal for K^+^ efflux.^110^ A second example is to establish a proton gradient, by applying a protonophore, such as Carbonyl cyanide m-chlorophenyl hydrazone (CCCP). In this instance the movement of protons into the liposome drives the efflux of K^+^ ions. The increase in proton flux may be measure by the quenching of a proton-sensitive fluorescence dye (Figure 4(A)).^72^ In both cases ligand may be added to investigate the impact on ion channel activity.

Single-channel patch clamp electrophysiology remains a principal approach for studying ionic flux (Figure 4(B),(C)). Ligands, such as PIP_2_, may be applied to an excised patch, with their influence on the single-channel properties recorded. However, application of lipids directly to the channel faces solubility and concentration issues. One of the more straightforward means to overcome the solubility issues of PIP_2_ is to use diC8-PIP_2_, which has shorter 8-carbons acyl tails, rather than the native 1-stearoyl-2-arachidonyl acyl chains. Di-C8-PIP_2_ has been used to calculate the EC_50_ value PIP_2_ binding to K_ir_3.2.^106^, ^111^ This short-tail lipid was also crystallised with the K_ir_2.2 and K_ir_3.2 channels detailed earlier in this review.^86^, ^87^

An alternative approach is to add molecules that compete for the phospholipids in the membrane. Previous studies have applied ATP as a PIP_2_ competitor to the channel, and therefore used this as a metric to measure binding.^1^, ^37^, ^38^, ^112^ Alternatively, the cationic neomycin may be applied to directly bind to PIP_2_ and therefore inhibit lipid-protein interactions through PIP_2_ sequestration.^113^ This thereby reduces the free concentration of PIP_2_ available to bind and activate an ion channel (Figure 4(C)).^114^, ^115^ This method provides an advantage over di-C8 in terms of the relative solubility of neomycin, and has been used as a tool to screen for the PIP_2_ binding site on K_ir_6.2 channels.^42^ Later work has used neomycin to remove the background signal from PIP_2_ activation on TASK2 K_2P_ channels in single channel recordings,^116^ or just simply to deplete the free PIP_2_ within the bilayer.^117^

Protein-lipid interactions have also been studied *in vitro* with spectroscopy methods, using a fluorescence PIP_2_ lipid analogue. Here, the fluorescent PIP_2_ analogue was used to measure competition at the lipid binding site. As the labelled PIP_2_ binds to a protein fused to GFP, FRET occurs between the lipid and protein fluorophores. By titrating in other lipids, to induce competition, the affinity of the lipid for the channel can be measured relative to the labelled PIP_2_^72^ (Figure 4(D)). Direct measurement at the lipid binding site is also currently possible using isotopic labelling of residues. This allows binding kinetics of weaker binders (mM range) to be quantified using NMR spectroscopy.^118^

Native mass spectrometry (nMS) has emerged as a technique to characterise the interactions of membrane proteins and lipids.^119^, ^120^ By recording ligand-binding curves of lipids titrated into a purified membrane protein sample, binding affinities can be assessed, as well as quantification of the number of putative lipid binding sites^121^, ^122^, ^123^ (Figure 4(E)). In addition, nMS experiments are employed to investigate the effect of lipids on protein oligomeric state and their stability.^121^, ^122^ Early nMS work on ion channel lipid interactions focused on the effect of lipids (and other effects) on the stoichiometry of the mechanosensitive channel of large conductance (MscL) in different bacteria.^121^ Furthermore, nMS has been applied to characterise the selectivity of lipid binding to the isoforms of the TRAAK channel^80^ and to K_ir_3.2 where PIP_2_ was shown to bind selectively and single mutations influence the affinities and specificity.^124^ A more comprehensive review on characterising protein-lipid interactions through nMS can be found in.^120^ Recent advances in sample preparation have enabled the recording of mass spectra of membrane vesicles, allowing interactions to be investigated in their potentially most native state.^125^, ^126^

The use of fluorescence has extended to investigate protein-lipid interaction and clustering under super-resolution fluorescence microscopy such as direct stochastic optical reconstruction microscopy (dSTORM).^127^ By using a lipid specific dye, the size and the intensity of the lipid specific fluorescence can be quantified to infer PA clustering (Figure 4(F))^128^. Through the labelling of specific proteins and lipids, it is possible to quantify colocalization of proteins and lipid, allowing the extent of their interactions to be investigated.^129^, ^130^

To couple channel activation to the binding process, patch-clamp fluorometry has been introduced as a method to record both current (channel activation/inhibition) simultaneously with the binding kinetics (Figure 4(G)). An extensive review on the method is noted in.^131^ Initially, the method was implemented to investigate K^+^ channel conformational change, by measuring the change in the fluorescence signal of the labelled protein.^132^, ^133^ The method is then applied to investigate protein–ligand interactions where both protein and ligand are labelled. By modifying one of the amino acids near the ligand binding site to 6-propionyl-2-(N,N-dimethyl)aminonaphthalene (ANAP), this allows ATP analogue such as trinitrophenol-ATP (TNP-ATP) to quench to fluorescence signal on the ANAP.^134^, ^135^ As the binding of PIP_2_ influences the binding kinetics of TNP-ATP, this method indirectly assesses the affinity of PIP_2_ to its binding site.^40^ This simultaneously yields the ligand binding constant and the kinetics of ion channel gating.

## Lipid binding and disease

Amino acid mutations within a lipid binding site have the potential to cause physiological defects to an ion channel and lead to clinical symptoms.^6^, ^8^ Mutations near the PIP_2_ binding site of K_ir_6.2 are associated with problems in insulin secretion.^8^ In the pancreatic β-cell, insulin secretion is triggered when the K_ATP_ channel is closed.^6^ Thus, mutations which alter channel opening cause defects in insulin secretion, for example mutations which enhanced PIP_2_ binding (such as K39R, E179A, E179K) are associated with neonatal diabetes (ND), whereas mutations which destabilise PIP_2_ binding (such as K67N) to the channel results in congenital hyperinsulinism (CHI) – an opposite phenotype.^8^, ^42^, ^136^, ^137^ Mutations to the PIP_2_ binding site of K_ir_2.1 (such as R218Q/W) are shown to be associated with Andersen-Tawil syndrome (ATS) which displays cardia arrythmia, periodic paralysis and dysmorphic features. Similar to K_ir_2.1, the K_ir_1.1 mutation at R312Q/W displays Bartter syndrome which results in polyhydramnios, premature delivery, hypokalemic alkalosis, and hypercalciuria.^41^

Mutations of the KCNQ channel are associated with QT syndrome, causing a longer QT hyperpolarised wave in the ECG signal.^7^, ^138^ Some of these mutations, e.g. R555H, are shown to be associated with PIP_2_ binding site. By understanding the consequences of PIP_2_ binding on the conformational changes of the protein, one can better appreciate how mutations are linked to clinical symptoms. In some cases this may also aid in the development of novel therapeutics, with a number of small molecule drugs known to bind to ion channel lipid binding sites or mediate allosteric coupling between PIP_2_ and the pore, e.g. CP1 which binds to the S4-S5 linker of the KCNQ1,^57^ or retigabine which binds to the pore domain and promotes channel opening through PIP_2_ mediated interactions.^139^

## Future perspectives and conclusions

As further lipid-bound ion channel structures become available, from X-ray crystallography and the multiple conformational states captured in the class-averages from cryo-EM, it is important to understand how lipids induce conformational change and to then relate this to function. A direct linear motion between two structural states of a protein is unlikely to reflect the true nature of its conformational transition, which might bypass certain short-lived intermediate states. Therefore, molecular simulations on a longer timescale and/or enhanced (accelerated) sampling may be used to allow transition events to be mapped between these states. In addition to striving for longer molecular simulation timescales, it is also important to be able to simulate larger assemblies to reach the dimensions of the current resolution limits of super-resolution microscopy. Of note, recent studies with super-resolution microscopy have revealed that clustering of lipids in the bilayer is driven by membrane protein localization,^140^ with comparable clustering observed using MD simulations.^141^, ^142^

A further bottleneck in studying protein-lipid interaction is the determination of their binding affinity. MD simulations provide relative affinity between protein and lipids based on the structure available, but the challenge is to compare those to experimental data. Novel approaches in mass spectrometry and the use of fluorescence-based analogues provide values for comparison with the computationally calculated binding free energies. Comparative studies between methods would highlight the significance of lipid binding, modulation of ion channel activity and separation between lipid binding and signal transduction.

One of the first papers directly relating the binding of PIP_2_ to the activity of K^+^ ion channels was published in 1996.^1^ 25 years later, we now have atomic level details of binding sites of PIP_2_, how PIP_2_ induces conformational change, the impact on single-channel kinetics and details of its potential roles in disease. Together this provides an interdisciplinary framework for understanding lipid modulation of K^+^ channels, which may be extended to membrane proteins in general.

## CRediT authorship contribution statement

**Tanadet Pipatpolkai:** Conceptualization, Investigation, Methodology, Visualization, Writing - original draft, review & editing. **Daniel Quetschlich:** Conceptualization, Investigation, Methodology, Visualization, Writing - original draft, review & editing. **Phillip J. Stansfeld:** Conceptualization, Investigation, Methodology, Visualization, Supervision, Funding Acquistion, Writing - original draft, review & editing.
